# The Dynamics of Youth Employment and Empowerment in Agriculture and Rural Development in South Africa: A Scoping Review

**DOI:** 10.3390/su14095041

**Published:** 2022-04-22

**Authors:** Wendy Geza, Mjabuliseni Simon Cloapas Ngidi, Rob Slotow, Tafadzwanashe Mabhaudhi

**Affiliations:** 1 Centre for Transformative Agricultural and Food Systems, School of Agricultural, Earth and Environmental Sciences, University of KwaZulu-Natal, Private Bag X01, Scottsville, Pietermaritzburg 3209, South Africa; 2 African Centre of Food Security, School of Agricultural, Earth and Environmental Sciences, University of KwaZulu-Natal, Private Bag X01, Scottsville, Pietermaritzburg 3209, South Africa; 3 Department of Agricultural Extension and Rural Resource Management, School of Agricultural, Earth and Environmental Sciences, University of KwaZulu-Natal, Private Bag X01, Scottsville, Pietermaritzburg 3209, South Africa; 4 Centre for Transformative Agricultural and Food Systems, School of Life Sciences, University of KwaZulu-Natal, Private Bag X01, Scottsville, Pietermaritzburg 3209, South Africa; 5 International Water Management Institute (IWMI-GH), West Africa Office, Accra GA015, Ghana

**Keywords:** agriculture participation, empowerment, government programmes, employment, unemployment, youth inclusion

## Abstract

Over the years, South Africa has made significant investments aimed at transforming the agricultural sector to deliver on rural economic development and job creation. These investments have had varying levels of success; still, what is worrying is the high youth unemployment rate which is amongst the highest globally. We conducted a scoping review using the PRISMA-P guidelines to identify the challenges youth face in accessing sustainable employment in the agriculture sector. Peer-reviewed studies were retrieved from online databases (Web of Science, Cab Direct, and Science Direct) for 1994–2021. The findings showed that youth are still facing significant challenges in the demand and supply side of the labour market and lack of inclusivity in policy formulation and implementation, limiting their involvement in agriculture and rural development initiatives. Policies and strategies responding to these challenges exist, and the spectrum of support services provided are primarily focused on entrepreneurship. Yet, the implementation of programs and initiatives has not been successful. This could be attributed to the obstacles persisting in the sociopolitical environment in SA, causing additional barriers to program implementation. Therefore, to enhance youth involvement in agriculture and rural development, there is a need to connect more rural youth to support services, local employment programmes, and youth inclusion in policy formulation processes. Additionally, the focus of policy and programs should be broadened to cater to different youth knowledge and skill profiles.

## 1 Introduction

As of the third quarter of 2021, the unemployment rate in South Africa (SA) reached a new record of 34.9%, increasing by 0.5% from the second quarter of 2021 [1]. The country currently has a youth unemployment rate of 66.5% [1], which is still among the worst globally [2,3]. The SA labour market is more favourable to men than women. The proportion of men in employment is higher than that of women, and the unemployment rate among men is lower than among women [1]. The unemployment rate among women was 37.3% in the third quarter of 2021 compared to 32.9% among men [1]. Yet the South African population is female and young. Despite assurances of a better life in the post-apartheid era, the majority of the ‘born free’ generation of young South Africans continue to face high levels of poverty, unemployment, and limited opportunities for upward socioeconomic mobility experienced by their parents decades earlier [4–7]. Because of the legacy of apartheid, rural areas in SA are marginalized, underdeveloped, and distant from economic advantages and opportunities [7]. Thus, youth in rural areas face poorer employment prospects and migrate to cities to seek opportunities [8]. Agriculture, which is envisioned as a vehicle for rural economic development, could potentially turn this tide by creating employment opportunities for rural youths where they reside.

A South African’s likelihood of being trapped in poverty is primarily determined by gender, race, and location [5,9,10]. Similarly, evidence emphasises the quality of education, socioeconomic background, policy design and implementation, the structure of the labour market, and lack of youth inclusivity as the main areas where challenges still exist [8,9,11–13]. Consequently, the combination of these factors produces a young workforce that is ill-prepared to compete in the economy, reinforcing spatial and gender inequalities, poverty, and unemployment [6,12]. Nonetheless, household-level barriers such as geographical isolation, low levels of social capital, lack of access to information, and the cost of seeking work hinder youth’s employability [7,14]. Furthermore, since the COVID-19 pandemic began, skilled employment has been much more durable than unskilled and semiskilled jobs [15]. Thus, many unskilled youths have lost their jobs, adding to the already high numbers of unemployed youth.

Identifying and addressing the main drivers of poverty, inequality, and unemployment amongst the youth is essential for supporting them to reach their potential and contribute to economic growth and sustainable development [16]. A holistic approach to youth employment and empowerment is required to address the challenges affecting youth participation in the labour market [13]. Agricultural transformation in lower- and middle-income countries is essential in supporting emerging economies, eliminating poverty and hunger [17]. Furthermore, food systems outcomes influence human nutrition, food security, health, environmental, social, and economic results [18]. Unlocking opportunities in the food system, however, would necessitate the creation of decent jobs for young people by supporting them to build skills relevant to the job market while also improving income security [19].

It is also critical to identify food-system-related challenges to unlock opportunities for livelihood-enhancing strategies [20,21]. Green jobs have the potential for job creation, skills development, and new opportunities for youth to start niche businesses and ensure better quality jobs within the value chain [22]. However, they require a higher skill level, dedicated and structured training, and coordinated policy frameworks to develop the ‘green skills’ required [19,23,24].

### Youth Empowerment in Agriculture

The youth empowerment element in policy is essential for accountability and reflects a commitment to support the enabling conditions that assist youth in taking charge of their own lives and well-being [25]. Jennings et al. [26], describe empowerment as a multilevel construct that can occur at multiple levels (e.g., individual, family, organization, and community). It includes social action processes, practical approaches, and applications that aim to increase control and mastery for improved equality and quality of life [26]. Equally, Martínez et al. [27], define youth empowerment programme activities as interventions that, based on young people’s strengths, involve them in decision-making processes regarding the design, planning, and implementation of the programmes themselves, and award them an active, central role. The concept of empowerment can also be linked to power, participation, and education [28]. Moreover, government youth empowerment programs influence the development paths that youth can take [29]. Therefore, solid youth empowerment in agriculture ought to support the efforts to increase youth participation in agriculture [30].

However, the apartheid regime caused inequalities in the spectrum of skills relevant to the agriculture sector in SA, particularly regarding Agricultural Education and Training (AET). To address these challenges, various initiatives and programmes were introduced post-1994 as part of a transformation agenda [31]. This included increasing support to AET through supporting Agricultural Colleges and Universities of Technology, the external bursary scheme, introducing the Young Professional Development Programme (Internship) in 2004, and the master mentorship programme [32]. Moreover, to promote equal participation in the sector, other skills development programs were implemented through AgriSETA, extension services, and agricultural finance schemes through the Land and Agricultural Bank and Micro Agricultural Financial Institutions of South Africa (MAFISA) [33]. However, despite these efforts to mainstream youth into the sector, support programs have achieved limited success [31] with numerous initiatives lacking consistent support, monitoring, and evaluation [34,35], including the impact of government corruption [36] and lack of coordination between land, agriculture, and rural development policies [37,38].

The long-term vision and strategic goals of SA are outlined in the National Development Plan (NDP) [39]. Its core vision is to reduce poverty and inequality while ensuring that all South Africans attain a decent standard of living. However, the goals of this long-term plan can only be realised if and when SA draws on the energies of its entire people, including the youth. The implications of this long-term plan are addressed in other strategies such as the Medium-Term Strategic Framework (MTSF) and the New Growth Path (NGP) [40]. Though, as noted by several authors [41–43], these policies lack the necessary mechanisms to effectively address the socioeconomic challenges they aim to resolve. For instance, the Expanded Public Works Programme (EPWP) [44] and the Community Works Programme (CWP) are programmes that are outputs of the NGP. These programmes have been designed and implemented to create employment for low skilled youth in SA. However, they have been associated with vulnerable working conditions, do not provide sustainable job creation in the long term, and do not provide skills development [45–48]. South Africa’s key policies such as the NGP and the NDP promote agriculture as a means to achieve all-inclusive growth, employment, and food security. In SA, agriculture accounts for 5% of the total employment [49] and can create more jobs rapidly, especially in rural areas where traditional industries are not incentivised to set up businesses. Thus, this scoping review investigates the challenges youth in democratic SA face to participate in the labour market, emphasising youth in agriculture and rural development. The specific objectives are to:

(a) Determine youth participation and empowerment in the agricultural sector in SA;
(b) Assess government policies, strategies, and programmes related to youth participation and empowerment in agriculture in SA to ensure equality and inclusivity of youth in the sector.

This review investigates the dynamics of youth employment and empowerment in agriculture and rural development in democratic SA. It begins by (i) exploring the primary factor determining youth participation in agriculture and shaping youth perceptions of the industry. Then, the review (ii) examines the main challenges experienced by youth in agriculture, (iii) describes additional factors preventing youth from securing sustainable employment in agriculture and rural development, (iv) addresses the main aspects to create an enabling environment for youth participation and empowerment, and lastly, (v) investigates policy focus and priority areas.

## 2 Materials and Methods

### 2.1 Literature Search

This desktop review was conducted using Arksey and O’Malley’s [50] methodological framework for scoping reviews and the PRISMA-P guidelines for conducting systematic reviews [51]. The literature search focused primarily on government policies, strategies, and programmes related to youth participation and empowerment in agriculture and rural development in South Africa from 1994 to 2021. This period covers the post-apartheid era and overlaps with the African Youth Decade plan of action [52]. Grey literature and other relevant policy documents (regional policies set by international government organisations) were retrieved from websites of key development organisations in Africa’s agriculture, for example, the Food and Agriculture Organization (FAO), African Union, Southern African Development Community (SADC), New Partnership for Africa’s Development (NEPAD), Institute of Development Studies, and United Nations agencies, among others. The review of policies, strategies, and programmes was complemented by a secondary literature search of peer-reviewed research articles using online databases, namely, Web of Science, Cab Direct, and Science Direct, based on studies conducted on youth in agriculture in South Africa published between 1994 and 2021.

The PCC (Population, Context, and Concept) nomenclature was adopted to determine the eligibility criteria for identified documents, and it was also used as a screening tool (see Table 1 below). In terms of population, the study included young people between the ages of 15 and 35, as defined by the African Youth Charter [53], who are involved in agriculture. Aside from agriculture involvement, the context also included young people in universities/agricultural institutions or any other agricultural training program. The literature search terms/keywords were ‘agriculture’ with the synonyms ‘farming’, ‘land management’, and ‘farm management’. The second keyword used was ‘participation’ with the synonyms ‘involvement’, ‘engagement’, and ‘contribution’. The third keyword used was ‘youth’ with the synonyms ’young people’, ‘adolescents’, and ‘young adulthood’. The keywords were used in combination with each other. The use of singular and plural and synonyms for search terms was also applied, accounting for relevant keywords that may differ from one database to another. For example, for the search terms ‘youth and agriculture’, ‘young farmers’, or ‘young people in farming’ were used.

After that, the peer-reviewed journal articles, policy and strategic documents were divided and analysed. The documents were separated to ensure that the content of larger documents did not skew or offset the analysis. As noted by Alves and Lee [54], a joint analysis tends to skew word frequency and word query results on qualitative data analysis software such as QRS NVivo, in favour of the lengthier documents.

### 2.2 Data Analysis

#### 2.2.1 Peer-Reviewed Journal Articles

For the peer-reviewed journal articles, the documents retrieved from the search were exported to the QRS NVivo 12 qualitative data analysis software [55]. A search query for the 20 most common words in the data set was conducted and word trees were generated (see Supplementary Materials, Figure S1). The number 20 was selected to give a snapshot of broad focused themes and connections within the data set related to opportunities and challenges for youth participation in agriculture. These were then translated into themes or ‘nodes’ for further analysis [55]. These nodes contained classifications such as ‘developmental initiatives’, ‘support required’, ‘youth characteristics’, ‘youth participation’, and ‘demographical challenges’. Further classification of the data set was carried out to code passages of the data under the appropriate node. Then, these nodes were further organized into cases based on topics. The topics included awareness of initiatives and programmes, youth interests and aspirations, mentorship, inclusion, training and experience, and entrepreneurship.

Then, to establish the factors determining youth participation and empowerment in agriculture, an explore diagram was generated to investigate the connections and links between the nodes and cases. The focal point of this explore diagram was the ‘awareness of initiatives and programmes’ case. Subsequently, a comparison diagram was created to show the relationships and similarities between negative and positive youth perceptions of agriculture. Afterwards, using the crosstab query and matrix coding functions within NVivo, aspects of the data set and results from the previous analysis were further analysed for patterns and connections. These patterns and relationships were explored to establish additional factors and challenges that youth face to secure sustainable employment in the agriculture and rural development sector.

#### 2.2.2 Policy and Strategic Documents

**Figure 1:**
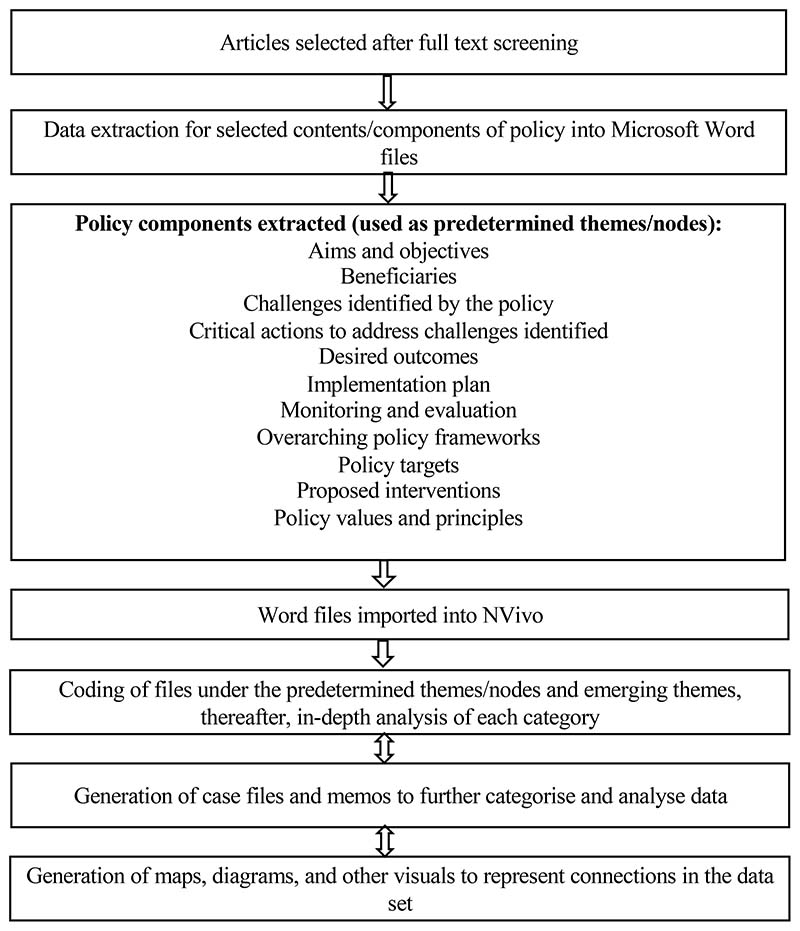
Data analysis process followed for policy and strategic document analysis.

The Content Analysis (CA) methodology was used in analysing strategic documents. Content analysis is a systematic method useful for analysing patterns, understanding context, and interpreting meaning in documents [56,57]. It is popular in the humanities and social science disciplines and has been used in analysing legislation [54,58,59]. In this study, CA was considered suitable for analysing trends and patterns in government policies, strategies, programmes, and youth empowerment initiatives in agriculture in SA. Although we argue the effectiveness of using CA in understanding the context of documents, we acknowledge the limitations of this methodology. There are risks associated with validity and rigour which may occur from possible personal biases [57]. To mitigate the risk of bias, two researchers initially performed the policy analysis separately, met to discuss their results, and obtained a consensus to increase validity. This was further reviewed by key experts in the field and thereafter reviewed by officials from the Department of Agriculture, Land Reform and Rural Development (DALRRD), and the Food Agriculture Organisation (FAO SA). Moreover, Figure 1 below maps the process followed during the analysis for transparency.

Deductive content analysis was used to explore the choices made about the policies’ content [60]. Based on the study’s objectives, the components of interest in policy and strategic documents are listed in Figure 1. Consequently, the main codes and themes for the analysis emerged from those components of interest. The contents of the policy documents from the areas of interest were extracted and pasted into Microsoft Word files for each record. These Word files were then exported to the QSR NVivo 12 qualitative data analysis software [55]. This allowed for a more in-depth interpretation of each component and avoided distortions that would arise and skew the software-aided analysis favouring the larger documents [54]. After that, the documents were coded under the predetermined codes, and new subcategory codes such as ‘priority areas’ and ‘distribution of resources’ emerged. Then, these nodes were further organized into cases based on topics. The topics included entrepreneurship, agriculture production, mentorship, creating an enabling environment, socioeconomic challenges, and inclusion. Once the coding was concluded, a more in-depth analysis of the categories was carried out to determine patterns, causal relations, and conducting word frequency queries to find the most frequently occurring words and concepts. These relationships were visualised using tree diagrams and graphs as per study objectives.

The first section of the results (cf., Section 3.1) presents the findings of the literature search and the characters of the studied articles. Section 3.2 presents the results on youth participation in agriculture and its associated factors. In Section 3.3, the challenges experienced by youth in agriculture in SA are presented. This also includes additional factors preventing youth from securing sustainable employment in the agriculture and rural development sector, whereas Section 3.4 of the study reports on elements to be addressed to create an enabling environment for youth participation and empowerment. Lastly, Section 3.5 reports on the government strategies and programmes related to youth participation and empowerment in agriculture and the proposed policy interventions and priority areas are outlined in Section 3.6.

## 3 Results

### 3.1 Literature Search Results

**Figure 2:**
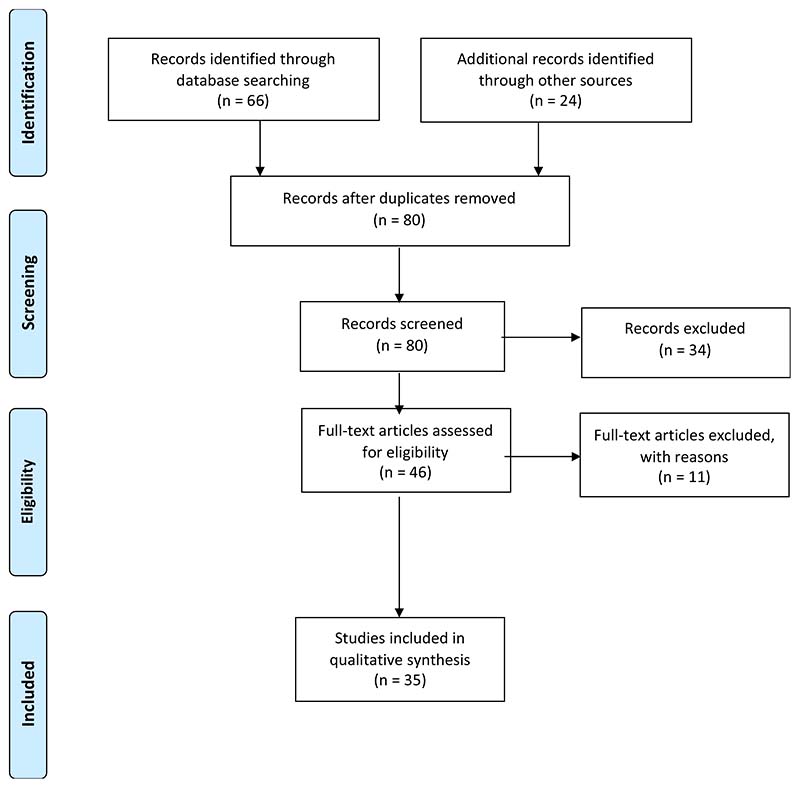
PRISMA flow diagram outlining protocol adopted in the scoping review based on the Preferred Reporting Items for Systematic Review and Meta-analysis Protocols (PRISMA-P) 2015 statement [51].

The PRISMA flowchart (see Figure 2) and the conduct and reporting of scoping reviews [61,62] were used as a guideline for reporting the review results. Although the literature search examined the period 1994–2021, no peer-reviewed literature published before 2006 was retrieved. In total, the literature search result found 80 studies after duplicates were removed. At the abstract screening stage, a total of 34 records were excluded, and 46 records were assessed for eligibility. From there, a total of 11 studies were excluded at the full-text screening stage. The most common reasons for exclusion were the lack of primary data and irrelevance towards research objectives. In the end, the analysis included a total of 35 documents (14 peer-reviewed documents and 21 policy documents), see Supplementary Materials, Tables S1–S3, for a summary of included and excluded documents. Most of the peer-reviewed documents were studies conducted in the Limpopo province (*n* = 5), followed by KwaZulu–Natal (*n* = 3), Mpumalanga province (*n* = 3), and the Eastern Cape province (*n* = 2). Only one study [63], researched all provinces in SA. The policy documents included policies and strategies on agricultural education and training, generic youth development and empowerment, and agriculture development (finance, support services, and entrepreneurship) (see Supplementary Materials, Table S2).

### 3.2 Youth Participation in Agriculture

**Figure 3:**
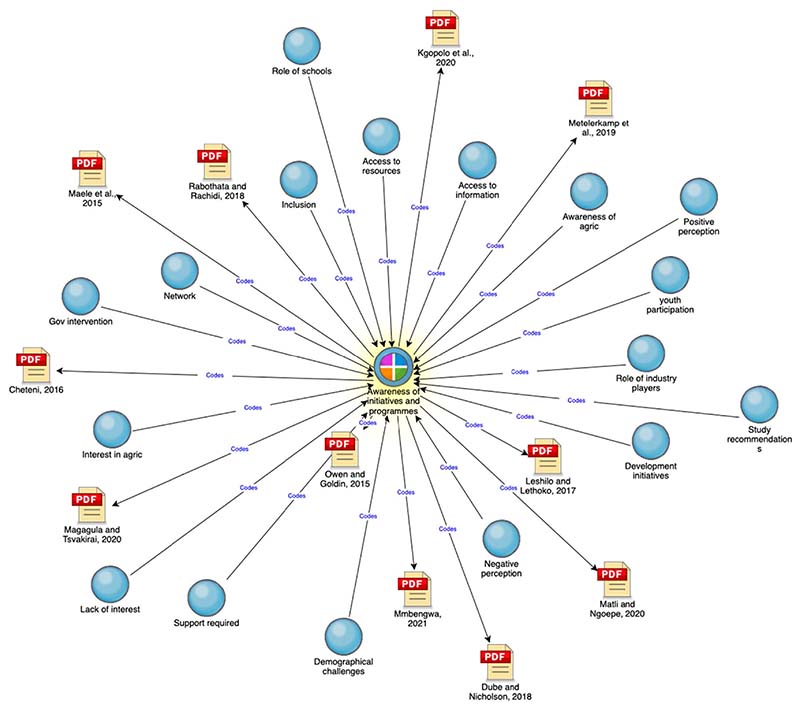
An explore diagram generated in NVivo to investigate the connections and links between the nodes (nodes based on pre-existing and emerging themes) and cases (cases based on topics). The focal point of this explore diagram was the ‘awareness of initiatives and programmes’ case.

**Figure 4:**
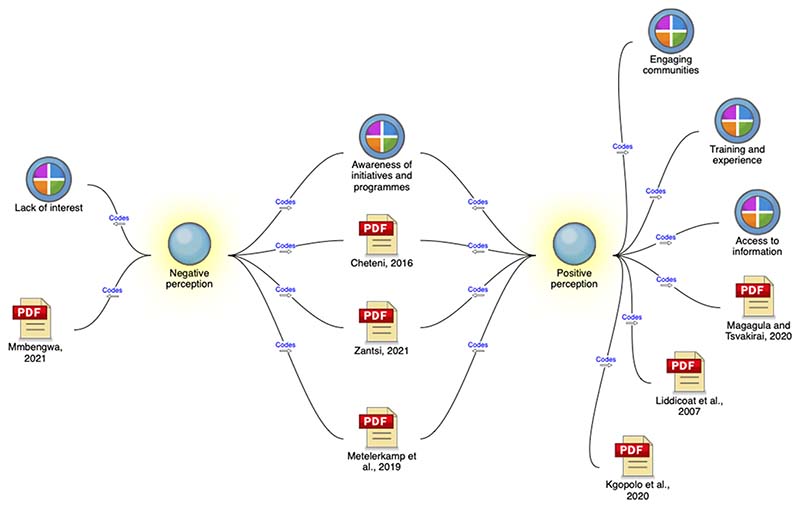
A comparison diagram showing the relationships and similarities between negative and positive youth perceptions of agriculture (extracted from NVivo).

According to the Youth Leadership Institute [64], youth engagement is the active, empowered, and intentional partnership with youth as stakeholders, problem solvers, and change agents in their communities. The analysis showed that the primary factor determining youth participation in agriculture and shaping youth perceptions of the industry is awareness of initiatives and programmes. As seen in Figure 3, awareness is fundamental as it is connected to participation, perception, aspirations, interest, and access to resources and information. It is also highlighted as a key recommendation from studies analysed. As shown in Figure 4, creating awareness of initiatives and programmes can be accomplished through various stakeholders such as schools, the private sector, government interventions, and development initiatives. However, awareness is also inhibited by demographical challenges such as being in remote rural areas [65], lack of social capital or having limited access to a network of people who are willing to share information [66–68], and access to the Internet and digital literacy [69]. This further marginalises youth in rural areas. Moreover, when comparing the difference between youths who have a negative perception of agriculture with those who have a positive perception of the industry (see Figure 4), awareness of initiatives and programmes is central in shaping youth perceptions.

### 3.3 Challenges Faced by Youth in Agriculture in SA

**Figure 5:**
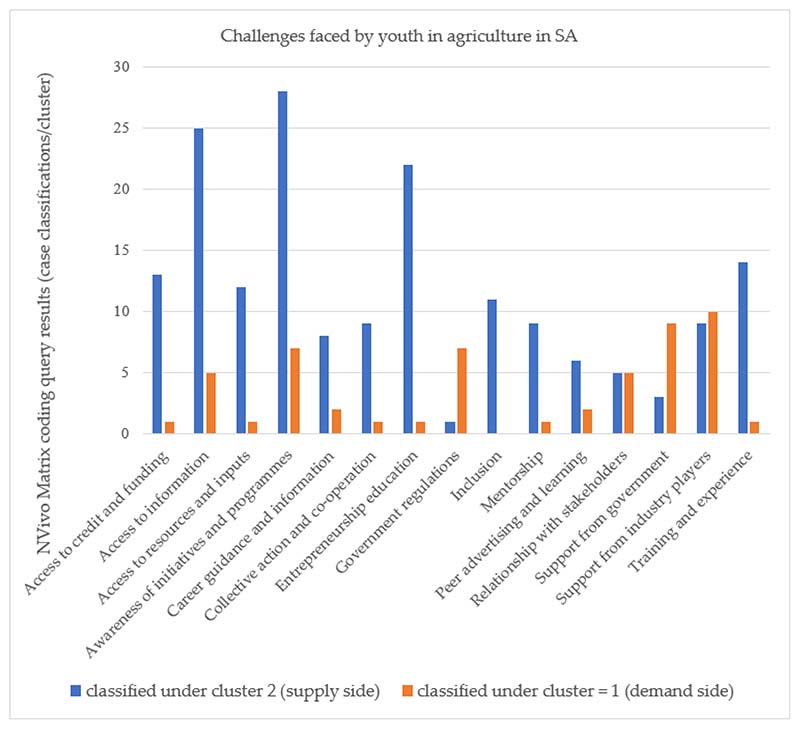
A graphical representation of challenges for youth in agriculture based on the analysis of issues stated in the studies included in this review. The main challenges were categorised into two broad clusters: the demand and supply side of the labour market.

Based on the analysis of issues stated in the studies included in this review, the main challenges experienced by youth in agriculture in SA can be categorised into two broad clusters: the demand and supply side of the labour market (see Figure 5). The demand side considers factors related to the labour market structure in a national and regional context [70]. The supply side refers to the features and characteristics of both the individual young person and their households [71]. These barriers can cause lower economic participation rates and other long-term negative consequences related to employability and well-being [72]. The factors categorised demand cluster are (i) government regulations, (ii) lack of a relationship with stakeholders, (iii) limited support from the government, (iv) limited support from private sector players, and (v) lack of youth inclusion in development programs, amongst others. The factors categorised supply cluster are (i) lack of access to credit and finance, (ii) lack of access to information, (iii) lack of awareness of initiatives and programmes, and (iv) lack of entrepreneurship education, amongst others.

The factors categorised under the demand and supply clusters regulate the economic environment conditions for youth in agriculture and those seeking employment in the industry. Moreover, the choices young people make in terms of their career interests, aspirations, and participation are a function of these factors and socioeconomic challenges summarised in Table 2. For example, as seen in Table 2, the lack of entrepreneurship skills [63,69,73] was a significant contributor in discouraging youth to pursue business. Other socioeconomic challenges included limited digital literacy skills amongst youth seeking employment [69], and the impact of low levels of education on entrepreneurial aspirations and success owing to limited literacy and numeracy skills [68,73]. Furthermore, the limited support for young farmers, unemployment, and lack of work experience contributed to the lack of interest in agriculture and entrepreneurship [36,74].

### 3.4 Creating an Enabling Environment for Youth Participation and Empowerment

**Figure 6:**
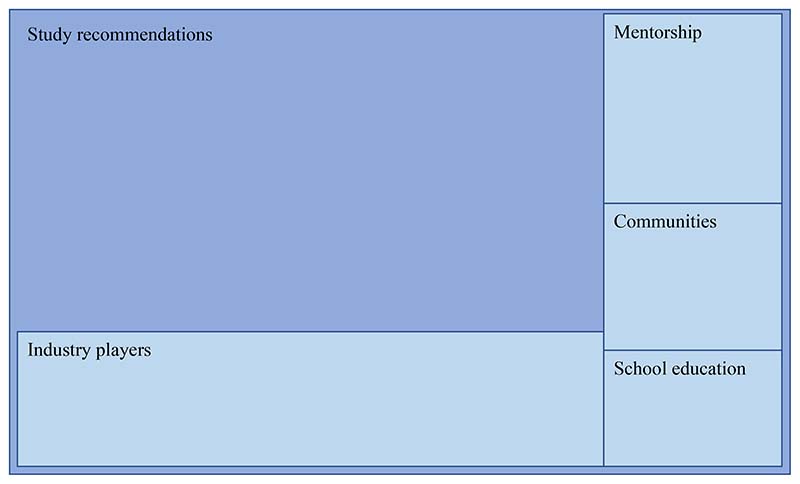
A hierarchy diagram generated in NVivo of the main themes presented as recommendations from studies analysed in the review.

The analysed studies presented four main reoccurring themes of aspects that need to be addressed to create an enabling environment for youth participation and empowerment. These themes, as shown in Figure 6, are (i) education, (ii) mentorship, (iii) stake-holders/industry players, and (iv) communities. Education is essential for providing career guidance information and awareness of agriculture careers at secondary school levels [65,74]. At the tertiary level, it is also essential for anchoring entrepreneurship skills and aspirations [63,69,73], whereas mentorship provides guidance and support as youth navigate their careers [65,75]. This support can also be provided by industry role players in creating awareness and sharing information related to initiatives and programmes available [65,73,75]. Moreover, social interactions in communities play a role in overcoming knowledge barriers, providing a network of support and communal learning to overcome social challenges [65,66].

### 3.5 Government Strategies and Programmes Related to Youth Participation and Empowerment in Agriculture

**Figure 7:**
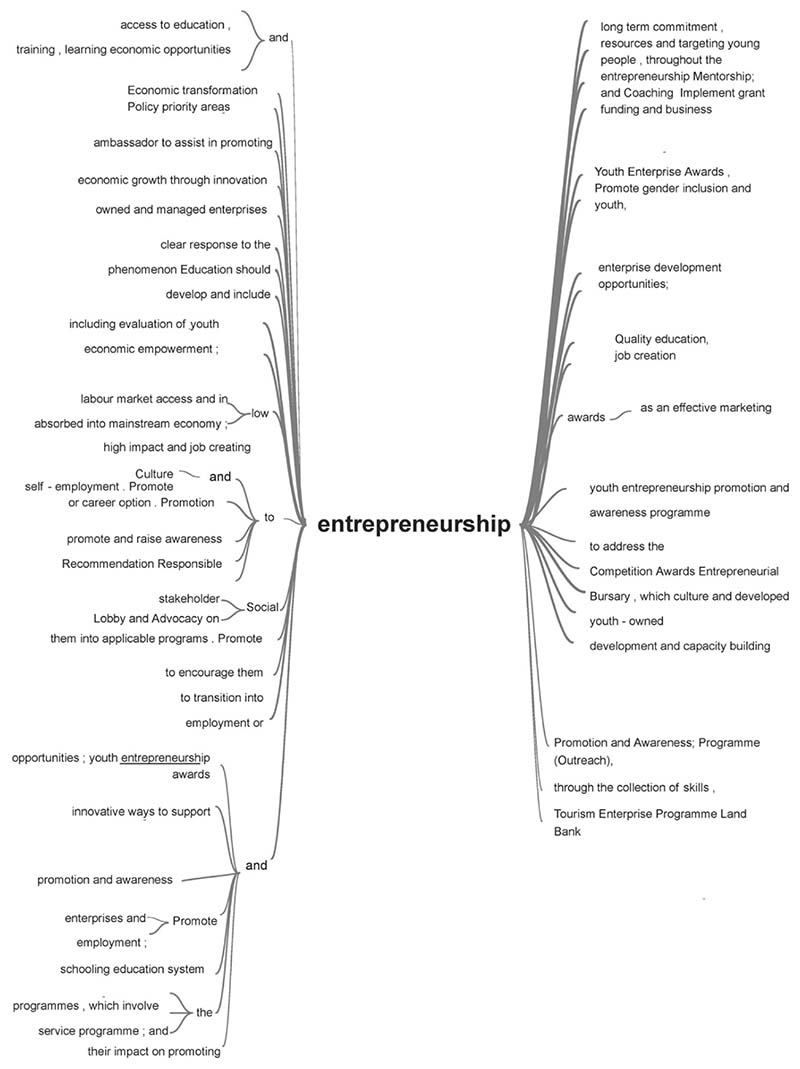
NVivo text search query results for the term ‘entrepreneurship’ within the data. Entrepreneurship emerged as a key topic classified under cases (second-order category).

The central focus of the strategy and policy documents included in this review is entrepreneurship. Entrepreneurship is the primary medium through which policy promotes economic transformation, stimulates youth inclusion in the economy, and creates jobs (see Figure 7). It is also the central theme in proposed interventions and support services, for example, youth enterprise development support, financial support services, awareness programs and initiatives, capacity development through education, regulations. Although the study’s focus is on youth participation in agriculture and rural development, most of the youth enterprise development support mentioned in policy includes other industries—for example, tourism, construction, infrastructure development, and social entrepreneurship. The emphasis on entrepreneurship also means that the support services being provided through policy and regulation, awareness campaigns, and incentives are mainly targeted at entry-level enterprise development and youth interested in owning businesses to encourage the creation of employment opportunities for their peers. Moreover, the overemphasis on the economic aspect undervalues other need to be addressed to facilitate youth participation and empowerment.

### 3.6 Proposed Interventions and Priority Areas

**Figure 8:**
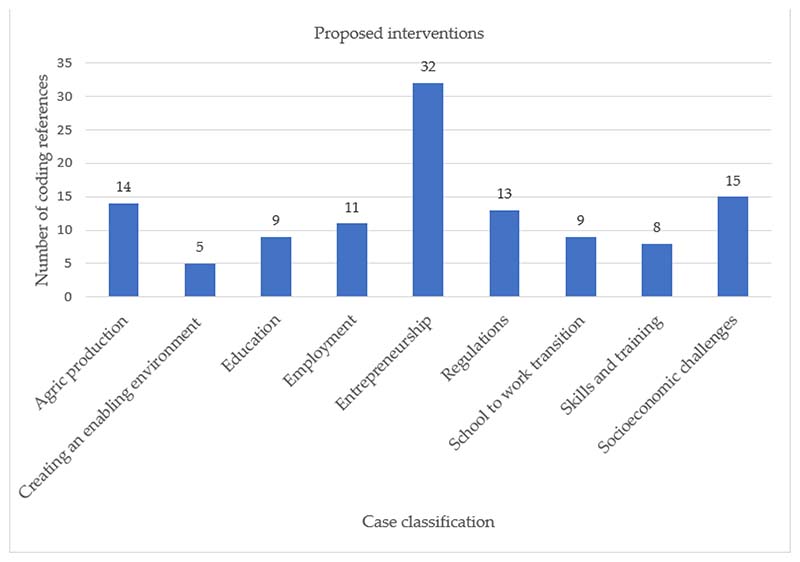
A graphical representation of the classification of proposed policy interventions (second-order category based on topics).

The leading ten proposed interventions are presented in Figure 8. The emphasis is again on (i) entrepreneurship, by developing and facilitating training workshops, programmes, and financial support for youth-owned Small, Medium, and Micro Enterprises (SMMEs) and cooperatives; (ii) addressing socioeconomic challenges that create barriers for youth to access the labour market, reducing levels of crime and violence, substance abuse, promoting health, well-being and HIV/AIDS prevention campaigns; (iii) improving agricultural productivity and increasing investments in support services such as rural infrastructure, storage and processing, markets, improving land ownership, and changing the image of agriculture as a career and livelihood choice amongst youth; (iv) promoting regulations and frameworks intended to support Black South Africans to actively participate fully in the agricultural sector as owners, managers, professionals, skilled employees, and consumers; and (v) creating sustainable job opportunities for youth through industrial growth.

Other proposed interventions include promoting better quality education through linking outcomes of school and tertiary institutions with labour market requirements; additionally, making career guidance a compulsory part of the schooling curriculum, improving access to education by providing multiple forms of education to meet the diverse needs of young people and the labour market, and developing the capacity of teachers and trainers. Furthermore, other interventions include developing youth skills through introducing training programs at local, regional, and national levels for entry into the labour market; creating an enabling environment for promoting youth economic empowerment and equipping young people to deal with socioeconomic challenges.

## 4 Discussion

From a social aspect, the legacy of apartheid laws and regulations left many people in rural areas in SA faced with poor economic prospects, inequality, unemployment, and poverty. Demographical challenges such as being in remote rural areas, lack of social capital, and limited access to the Internet remain prevalent in hindering youth’s ability to participate in the economy. Similarly, Graham et al. and Abay et al. [76,77] found that economic opportunities appear to be quite limited for many young people in rural areas, including graduates. Additionally, Wilkinson et al. [78] discovered that the three most common barriers to youth employment in Mpumalanga (SA) were lack of skills, lack of information on job openings, and overall lack of jobs. Youth are distributed across various economic landscapes, and their opportunities differ accordingly. Although since 1994 the government has made progress in policy formulation and planning, the youth are still faced with the same deteriorating socioeconomic challenges that hinder meaningful participation in the mainstream agriculture economy [31,79]. These socioeconomic challenges keep youth disconnected and excluded from opportunities that globalization and the changing political landscape may offer. Therefore, the heterogeneous landscapes in which youth exist should be considered and accounted for during policy design and implementation. This will ensure that policies are implementable in various contexts and are also responsive to context-specific challenges and opportunities for youth in agriculture. Thus, our results agree with Wilkinson et al. [78], that there is a need to connect more rural youth to local employment programmes that could be useful to them.

Although addressing socioeconomic challenges that create barriers for youth to access the labour market is reflected amongst the key proposed interventions in policy, the results suggest that the implementation has not been successful. Similarly, Diraditsile [29] also found that government youth empowerment programs in Botswana are well designed with admirable provisions for youth empowerment. However, the implementation is poor; officials have limited capacity and youth are barely involved in formulating and implementing interventions meant to benefit them.

The evidence suggests the need for multi- and transdisciplinary approaches. The co-dependency nature of the sustainable development pillars within various socioeconomic contexts requires commitment and partnerships between all key societal actors/stakeholders [80]. Stakeholders play a role in overcoming social and economic barriers and facilitating the necessary private–public and public–civil-society interactions needed to overcome challenges. For example, the results also indicate that creating awareness of initiatives and programmes can be accomplished through partnerships between schools, industry players, government interventions, and development initiatives. Youth awareness of initiatives and programmes is fundamental as it is connected to participation, perception, aspirations, interest, and access to resources and information. Moreover, improving youth’s awareness of and access to employment resources already available in their local area is essential to increasing their chances of participation in the economy [78].

This, however, may be challenging to achieve in SA. Sutherland [38], highlights the significant inconsistencies related to the roles assigned by the government to the private sector over time in general and sectoral policies. Challenges of incoherency in policy, poor economic policies, and lack of coordination in SA emerged as early as 1996, shortly after democracy [37]. Additionally, Mmbengwa et al. [81] argue that incubation programs that seek to attract youth to start their enterprises in SA are often under-resourced, riddled by financial corruption, and hindered by poor publicity. Social cohesion is essential for these partnerships to create awareness and increase youth participation in agriculture. Social cohesion builds a foundation for growth and development through promoting inclusivity and participation while encouraging strategies to reduce inequalities in the domains of activity (economic, political, and sociocultural) [82].

Nonetheless, Todes and Turok [83] caution that marginalised communities in SA are suspicious of state-sponsored initiatives and are impatient for tangible progress. Consequently, this causes disturbances and disagreements amongst stakeholders on project goals and priorities, ultimately preventing projects from proceeding. The evidence suggests a breakdown in trust between government and communities. By actively encouraging social cohesion and fostering partnerships between societal actors, the government can restore confidence in the community over time. Additional effort is also required to include youth and other society members through meaningful engagement, co-designing, co-implementation, and setting up community structures that can hold government officials to account for progress on community projects. This will aid in addressing the labour market barriers experienced by youth in agriculture by promoting inclusivity, creating opportunities for mentorship, access to information, entrepreneurship education, and improving the effectiveness of policies. Moreover, opportunities to participate in the economy and attain a decent standard of living will be improved for young South Africans.

From an economic aspect, entrepreneurship is the primary medium in which policy promotes economic transformation, stimulates youth inclusion in the economy, and creates jobs. Consequently, most of the support services provided through policy and regulation, awareness campaigns, and incentives are mainly targeted at entry-level enterprise development and youth interested in owning businesses. Very few programmes and interventions lead the youth into the mainstream agricultural economy. Whereas policy emphasis on youth involvement in nonfarm employment is important [84]. The agricultural sector offers other opportunities in nonfarm activities throughout the value chain [77]. However, programmes and interventions do not elaborate on how young people will be integrated into the value chain, nor do they extensively cater for educated and skilled youth seeking employment.

Nonetheless, the emphasis on the economic aspect also undervalues other enabling factors that need to be addressed to facilitate youth participation and empowerment. For example, the results highlight the lack of entrepreneurship skills and the impact of low literacy and numeracy skills on business success as key contributors in discouraging youth to pursue business, further emphasizing the importance of multi- and transdisciplinary approaches to address key enabling factors and barriers in youth empowerment and economic participation. For example, this can be achieved by integrating already existing policies such as the Agricultural Education and Training (AET) strategy, with policies focused on addressing challenges in the agriculture sector such as AGRI BEE. The joint effort of these policies would address challenges experienced by youth in agriculture while promoting better quality education, linking school outcomes with the job market’s needs, and providing career guidance. Additionally, increasing access to education through delivering multiple forms of education would meet the diverse needs of young people and develop the capacity of teachers and trainers.

## 5 Conclusions

This scoping review aimed to investigate the challenges youth in democratic SA face to participate in agriculture and rural development programmes. Specifically, the study investigated youth participation and empowerment in the agricultural sector in SA, and assessed relevant government policies, strategies, and programmes related to youth participation and empowerment in agriculture to ensure equality and inclusivity of youth in the sector. The results showed that, although progress has been made since 1994 towards promoting youth participation in agriculture, youth still faced significant challenges that limited their effective participation in agriculture. In general, youth are still faced with the same socioeconomic challenges that hinder meaningful participation in the mainstream agriculture economy. Moreover, these socioeconomic challenges perpetuate the exclusion of youth from opportunities that globalization and the changing political landscape may offer, including their awareness of opportunities, initiatives, and programmes targeted at youth. This is particularly true for rural youths, and those with low levels of education. This perpetuates a vicious cycle, which entrenches exclusion and inequality as opposed to inclusion and equity. Additionally, youth face obstacles in the demand and supply side of the labour market, lack of inclusivity in policy formulation and implementation, lack of entrepreneurship skills, receive poor quality education, and have limited available support for agripreneurs. Thus, the combination of these factors limits youth’s ability to participate in the labour market.

The evidence suggests the need for multi- and transdisciplinary approaches. All societal actors have a role in overcoming barriers to youth empowerment and participation in agriculture. Currently, the political and social climate in SA does create additional obstacles to the successful implementation of programs. South Africa needs better sectoral (public, private, and civil society) coordination and collaboration in planning policies to promote social cohesion that can facilitate implementation. This also includes improvements in the institutional capacity at all spheres of government for better program implementation.

Moreover, a holistic approach for interventions targeted at youth empowerment and promoting youth participation in the economy is recommended. Policies need to be informed from a broader perspective of sustainable development. This also requires additional efforts from the government to encourage collaboration between societal actors and stakeholders together with coordination and co-implementation between government sectors and departments to address socioeconomic barriers preventing youth from successfully participating in the economy as employers and employees. Additionally, the focus of government policies and programs should be widened to cater to youth’s diverse economic needs and career aspirations throughout the agricultural value chain. There is a need for holistic and systematic policies that focus on broader youth participation and empowerment in the food system other than just entrepreneurship. Furthermore, effort needs to be directed towards education and awareness as enablers that can facilitate youth participation in agriculture. Within the context of this review, education is essential for providing career guidance information, anchoring basic skills and aspirations towards agriculture activities and entry points that youth can choose to participate in. Awareness of opportunities, initiatives, and programmes in agriculture is linked to youth perceptions, aspirations, and interests. Furthermore, there is a need for increased effort to connect youth to support services already available in their areas.

## 6 Limitations

The results of the study should be considered in light of some limitations. Because of the search inclusion and exclusion criteria used for this study, other publications may be excluded. The search was limited to studies published between 1994 and 2021. This period covers the post-apartheid era. Furthermore, the selection of primary studies focused on programs or interventions that have been explicitly designed for youth between the ages of 15 and 35 years. Thus, data presented in this study are from papers relevant to the study’s objectives. Moreover, the articles retrieved during the search mainly conducted research on youth in rural areas. Therefore, the results presented in this study are biased towards youth in rural areas vs. youth in peri urban and urban areas. For future research, there is a need to broaden the scope of the research to ensure the inclusion of the full spectrum of themes related to youth and agriculture; however, this should consider the tradeoffs between breadth vs. depth.

## 7 Recommendations and Policy Implications

The results suggest that youth face numerous socioeconomic challenges that affect the type of economic opportunities they have access to, including their awareness of employment opportunities and programmes targeted at youth. Moreover, the focus of government policies and programs is narrow, with a lack of youth inclusivity in policy formulation and implementation, lack of entrepreneurship skills, poor quality education, and limited available support for agripreneurs. Thus, the combination of these factors limits youth’s ability to participate in the labour market. The implementation of government programs to address these issues has not been effective. Moreover, the political and social climate in SA does create additional barriers to the successful implementation of programs. Based on evidence found in this scoping review, this study therefore recommends:

◾ Investments need to be made to improve the institutional capacity at all spheres of government for better program implementation. Additionally, an effort is required to maintain the accountability and integrity of government in society. This is necessary for better program implementation and functional partnerships with the private sector, industry role players, and society. We need better sectoral (public, private, and civil society) coordination and collaboration in planning policies—collaboration and coordination will facilitate a social compact that can facilitate implementation.
◾ To better equip agricultural graduates, practical agribusiness training should be included in the undergraduate curriculum of agriculture qualifications. This can be achieved by providing support to implement policies such as the AET strategy together with upscaling capacity development programs such as Future Farmers Foundation and the Junior LandCare programme.
◾ There is a need to connect more rural youth to local employment programmes that could be useful. Additionally, ensure that youth in various socioeconomic contexts have access to opportunities advertised on online platforms such as AgriStaff, SA NGO pulse portal, puff and pass, and Career Junction.
◾ More effort is needed to connect youth involved in farming with support and initiatives available in their local areas. This could be achieved through partnership and advertisements by community structures, for example, schools, local clinics, churches, traditional authority councils, and established nongovernmental organisations with local communities. Alternatively, upscale existing support services such as Harambee and AFASA.
◾ The spectrum of support services provided through policy, regulation, and incentives should be broader and cater to different youth knowledge and skill profiles, other careers in the value-chain, and not just entrepreneurship. Moreover, for youth interested in entrepreneurship, the support provided should sustain long-term career growth and not only entry-level enterprise development. There is room for policies to focus on other forms of youth participation and empowerment other than just entrepreneurship. Moreover, there needs to be education and awareness around the various aspects of the agricultural sector, activities, and entry points that youth can choose to participate in.
◾ Young people must be active participants in all youth empowerment programmes, and the participation should recognise differences and inequalities between urban and rural youth and gender. More effort needs to be directed towards creating opportunities for youth (representative of all socioeconomic backgrounds) to participate actively in policy formulation, program design, and implementation.
◾ Policies need to be informed from a broader perspective of sustainable development, which understands that the economics are embedded within the social and environmental dimensions, all of which are underpinned by governance. Moreover, the results show that the issues that affect youth are systemic, and a broader focus beyond just the traditional understanding of agriculture is needed. This calls for a food systems approach as opposed to a specific focus on just agriculture. Furthermore, such an approach could also integrate with other systems.

## Supplementary Material

Supplementary information

## Figures and Tables

**Table 1:** The PCC (Population, Context, and Concept) nomenclature adopted to determine the eligibility criteria for screening and selection.

Include	Exclude
Population	
Young people in agriculture between the ages 15 and 35 years old Young people in universities/agricultural institutions between the ages 15 and 35 years old	Focus on males only Focus on females only
Context and Concept	
Interventions researching the participation or empowerment of youth in agriculture Intervention/study creating or identifying opportunities for youth participation in an agriculture program Studies researching youth perceptions, awareness, or attitude towards agriculture Program or intervention analysing social or political environment factors affecting the participation of young people in agriculture Interventions or studies conducting research that addresses a challenge or limitation faced by youth in agriculture Government policies, strategies, and programmes related to youth participation and empowerment in agriculture for South Africa from 1994 to 2021 Qualitative and quantitative studies	Literature reviews/review papers or studies with no primary data Studies not conducted in South Africa/not of relevance to South Africa Studies on food insecurity, diets, or nutritional status of youth Farm injuries in young workers Youth urban migration Studies focusing on gender gaps concerning youth employment challenges or opportunities

**Table 2:** Additional factors preventing youth from securing sustainable employment in the agriculture and rural development sector (adapted from NVivo crosstab results).

Nodes	Lack of Interest	Securing Employment	Support for Farmers	Total
Support required	0%	0%	60%	12.5%
Aspirations	0%	0%	0%	0%
Demographical challenges	0%	18.75%	40%	20.83%
Digital literacy	0%	50%	0%	33.33%
Education levels	33.33%	0%	0%	4.17%
Entcepreneurship	33.33%	0%	0%	4.17%
Gender	0%	0%	0%	0%
Unemployment	33.33%	31.25%	0%	25%
Total	100%	100%	100%	100%

The shading in the cells represents patterns and relationships in the matrix amongst factors preventing youth in securing; employment. The shade intensity increases with the percentage value.

## Data Availability

The data presented in this study are available on request from the corresponding author.
