# Supplementary material for: The Dynamics of Youth Employment and Empowerment in Agriculture and Rural Development in South Africa: A Scoping Review
Source: Sustainability. Author manuscript; Available in PMC 2023 Sep 8. (PMC7615068; doi:10.3390/su14095041)
Supplement: Supplementary information [file EMS187375-supplement-Supplementary_information_.docx]

**The dynamics of youth employment and empowerment in agriculture and rural development in South Africa: A scoping review**

**Supplementary information**


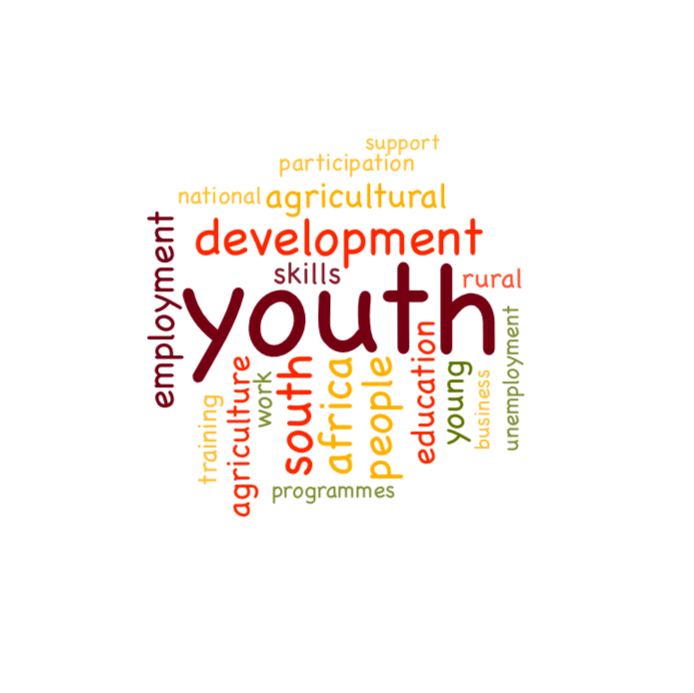


Figure S1: NVivo search query results for the 20 most common words in the data set. This figure provides a snapshot of broad focused themes within the data set.

Table S1: Characteristics of studies included in the review

| **Author and year** | **Title** | **Location of the study** | **Study aims** | **Context** | **Sample size** | **Main occupation**  **of the study participants** | **Study outcomes** |
| --- | --- | --- | --- | --- | --- | --- | --- |
| Cheteni (2016) | Youth Participation in Agriculture in the Nkonkobe District Municipality, South Africa | Nkonkobe Municipality, Eastern Cape | To assess factors influencing the participation of youth in Agriculture in Nkonkobe District Municipality in the Eastern Cape Province, South Africa. | Rural area | 140 | Secondary school students (73 percent) and temporary employed | The findings from the survey highlighted that youth are not participating in farming as highlighted by a number of research articles in South Africa. Many young people still view agriculture as working in farms physical |
| Dube & Nicholson (2018) | Youth in agriculture: securing the future of sugarcane farming in south Africa | KwaZulu-Natal | Aims to provide the benefits of promoting youth participation in the South African sugar industry; it also aims to identify existing programmes to support youth in agriculture in South Africa, while assessing the extent to which youth in agriculture programs promote future sugarcane farming in South Africa. | Rural and urban | 24 | Students, farmers and professionals in the agriculture industry | Most of the young people who participated in the survey were not aware of most of the initiatives available for them both at a National and Provincial level. Access to information, land and funding remains a barrier for young people to start and maintain agricultural enterprises. |
| Kgopolo, et.al (2020) | Youth Participation in Farming Information related Activities in Mahikeng Local Municipality, North West province South Africa | Mahikeng Local Municipality, North West province | investigating youth participation in farming related activities in Mahikeng North west province of South Africa. | Mainly rural | 120 | Not mentioned | The findings of the study revealed that young people hold a positive view about  agriculture and agricultural careers. The respondents were able to indicate the role that agriculture play in their life, in the community and in the country |
| Leshilo & Lethoko (2017) | The contribution of youth in Local Economic Development and entrepreneurship in  Polokwane Municipality, Limpopo Province | Polokwane Municipality, Limpopo Province | to investigate the contribution of the youth to local economic development and entrepreneurship | Mainly rural | 50 | entrepreneurs | The findings revealed that a lack of access to finance, entrepreneurship education and training, market opportunities and an awareness of the initiatives offered by government all pose significant obstacles to young entrepreneurs, who are additionally constrained by stringent government policies and regulations |
| Liddicoat et.al, (2007) | Sharing Programs across Cultures: Lessons from Garden Mosaics in South Africa | Durban, KwaZulu-Natal | Garden Mosaics is a U.S.-based youth and community program that incorporates youth participation and engages young people in learning from elders, many of whom are immigrants, about growing food in urban gardens. In 2005, we adapted the Garden Mosaics activities for learners in two South African township schools. | Townships | 86 | Primary and secondary school students | It became clear that although Garden Mosaics is designed for use in multicultural settings, incorporates intergenerational learning, draws on local knowledge, addresses a widely relevant issue, and was intended to be adapted, it was not fully usable in a new context. |
|  |  |  |  |  |  |  |  |
| Maele et.al, (2015) | Characterization of Youth Agricultural Projects in Limpopo Province of South Africa | Limpopo Province | This study was done with the purpose of characterizing participants in youth agricultural projects and subsequently proposing strategies for increasing their productivity. It focused on youth agricultural projects supported by the Limpopo Department of Agriculture | Mainly rural | 50 | youth agricultural project beneficiaries | Important constraints faced by youth agricultural projects included (1) weak relationship between youth projects and other stakeholders resulting in limited access to information, (2) lack of access to funding, and (3) poor land tenure. Youth projects had strong relationships with youth cooperatives, and those should be used to disseminate information to the projects. |
| Magagula & Tsvakirai (2020) | Youth perceptions of agriculture: influence of cognitive processes on participation in agripreneurship | Mpumalanga Province | Examining the nature and key indicators that inform the perceptions of the youth towards the agricultural industry, and investigating the eﬀects of these perceptions, as well as other socio-economic factors impacting on the intentions of youth towards participating in agricultural business ventures. | Mainly rural | 120 | Students | The societal embeddedness of the agricultural industry is an equally important guide to people’s intention to start businesses, in addition to the proﬁt maximisation aspirations. |
| Matli & Ngoepe (2019) | Capitalizing on digital literacy skills for capacity development of people who are not in education, employment or training in South Africa | Gauteng province | The general purpose of the study was to explore how digital literacy skills can capacitate people who are NEET. | townships and urban areas | 9 | NEET youth ( not in education, employment or training) | The results indicate that digital literacy skills are essential when looking for employment opportunities. it is equally important for government to work collectively with non-governmental organizations (NGOs) and other stakeholders to establish policies that drive the development of NEET people in literacy skills. |
| Metelerkamp et.al, (2019) | We’re ready, the system’s not – youth perspectives on agricultural careers in South Africa | the Western Cape, KwaZulu-Natal and Limpopo Province | this paper seeks to provide an empirical foundation for understanding youth attitudes towards agriculture in South Africa | rural areas | 573 | Fifty four per cent had never been employed or run their own business. | Results showed that attitudes towards careers in agriculture varied greatly. While a set of negative perceptions emerged from the narratives as anticipated, over one third of respondents expressed a clear interest and passion for agriculture. This interest persisted in spite of a range of pervasive social norms and stigmas. This raises the need to begin questioning the commonly accepted truth that youth are not interested in agriculture. |
| Mmbengwa et al., (2021) | Determinants of youth entrepreneurial success in agribusiness sector: the case of Vhembe district municipality of South Africa | Vhembe district, Limpopo province | To identify the factors influencing youth agricultural entrepreneurship success in South Africa to reduce the ever-growing youth unemployment in South Africa with a particular reference to youth in Vhembe District Municipality. | Rural area | 325 | youth entrepreneurs who are smallholder farmers | Evidence from the study suggests that perseverance and personal motivation have the most significant influence, followed by creativity and a positive attitude. Secondly, South Africa’s youth in general and in Vhembe, particularly, associate agricultural enterprises as poor man’s business and thus see it as unattractive. |
| Mudau &  Kruger (2014) | Students Perception on the Entrepreneurship Curriculum at the University of South Africa’s Department of Agriculture and Animal Health | The study comprised all nine provinces in South Africa. | This study proposes the following research  questions: (1) How do Unisa’s agricultural students perceive entrepreneurship curriculum towards their post graduate innovation and work platforms?; (2) Does entrepreneurship curriculum have an impact on students?; (3) How can the College of Agriculture and Environmental Sciences be more effective in their provision training and assessment towards students entrepreneurship skills? | Mainly rural | 200 | agri-entrepreneurs and post graduate students | In the context of this study, it is clear that the  curriculum presented at Unisa’s CAES: Department of Agriculture and Animal Health is not appropriately developed to prepare students for entrepreneurship as a career option. |
| Owen & Goldin (2015) | Assessing the relationship between youth capabilities and food security: a case study of a rainwater harvesting project in South Africa | Mpumalanga Province | The research aimed to determine the capabilities that exist  amongst the youth, the state of food security in the villages, whether there is a relationship between food security and youth capabilities, and which capabilities of youth would best promote food security, and to better understand the differences that emerge among the youth around issues of food security and capabilities in the two villages. | rural villages | 199 | Not mentioned | The critical point of this paper is that youth have enormous potential to do and be more than they are actually doing and being in relation to food and water resources management – and that engaging them in food and water security projects more deliberately would provide them with more freedoms to be and to do what they value to be or to do. |
| Ramohale & Rachidi (2019) | Involvement of Unemployed Graduate Youth in SMME Support Programmes: The Case of Mankweng Area, Limpopo Province | Capricorn District Municipality in the Limpopo Province | This article presents data on the rate of youth unemployment in South Africa, the role that SMMEs can play in alleviating unemployment, and the support measures that are available for youth-owned SMMEs. | township | 15 | graduates, unemployed and looking for work | The results indicated that youth are not aware of the support programmes, and they have no intention of starting their own businesses. The lack of awareness of support programmes suggests that these programmes are not visible enough, especially to youth in rural areas, in this case, Mankweng area, who may need them the most. |
| Zantsi (2021) | Why do agricultural co-operatives fail to attract youth and create rural employment? Evidence from a case study of Zanokhanyo in Butterworth, Eastern Cape | Butterworth, Eastern Cape | This article attempts to give insight into why the Zanokhanyo Food Security agricultural cooperative has failed to provide employment and attract youth. | rural villages | 18 | Seeking employment | With cooperatives such as ZFSC, the idea of attracting youth and increasing rural income will hardly be achieved unless intensive production measures are implemented, and competent training is given to the members. |

Table S2: Strategies, reports and policy documents included in the review

| **Organization/ institution** | **Documents name** | **Policy aims and objectives** |
| --- | --- | --- |
| African Union | African Youth Charter | The aim of the African Youth Charter is to recall the historic injustices imposed to Africa such as slavery, colonization, depletion of natural resources, and considering the firm will of African people ‘s self-determination and the economic integration of Africa. |
| African Union | SADC Regional Agricultural Plan (RAP) | The primary aim of the policy is to document the objectives, support actions and desired outputs to achieve the SADC Common Agenda. The SADC Common Agenda seeks to stimulate equitable and sustainable economic growth whilst promoting socio-economic development at national and regional level, and consequently contributing towards regional integration. |
| Buffalo city Metropolitan municipality | Buffalo city metropolitan municipality child and youth development strategy 2017 - 2021 | The aim of this policy is the development of children and youth aligning with Chapter 10 of the Constitution of the Republic of South Africa Act 108 of 1996 which promotes an integrated, cooperative, and developmental local government inclusive of children and youth in accordance with the accompanying rights, responsibilities, and obligations |
| Department of Agriculture, 2001 | Strategic plan for South African Agriculture | The vision for the strategic plan for South African Agriculture is to have a united and prosperous agricultural sector. The implications of this vision are there must be a sustained profitable participation in the South African agricultural economy by all stakeholders, recognising the need to maintain and increase commercial production, to build international competitiveness and to address the historical legacies and biases that resulted in skewed access and representation. |
| Department of Agriculture, 2004 | Black Economic Empowerment framework for agriculture (AgriBEE) | To pursue Broad-based Black Economic Empowerment in support of a United and Prosperous Agricultural Sector |
| Department of Agriculture, Forestry and Fisheries | Comprehensive Agricultural Support Programme (CASP) | The aim of (CASP) is to enhance the provision of support services to promote and facilitate agricultural development targeting the beneficiaries of the land and agrarian reforms. |
| Department of Agriculture, Forestry and Fisheries (DAFF), 2014 | Agricultural Policy Action Plan | The aim of the APAP is to address growing concentration within the market which impacts job creation, transversal action programmes seek to localise food networks, through infrastructure development, incentivising support for SMMEs and small-scale producers across agriculture, forestry and fisheries value chains. |
| Department of Agriculture, Land Reform and Rural Development | National Policy on Comprehensive Producer Development Support | The main objective of the Policy is to regulate and guide the provision of support measures to the various categories of producers, thereby contributing to the restoration of the natural resources, a sustainable and competitive agricultural sector. It (StatsSA, 2021, Moher et al., 2015)aims to regulate and guide numerous interventions (support services) provided to the various categories of producers by government. |
| Department of Agriculture, Land Reform and Rural Development (DALRRD) | National Policy on Comprehensive Producer Development Support | To regulate and guide the provision of support measures to the various categories of producers, thereby contributing to the restoration of the natural resources, a sustainable and competitive agricultural sector. |
| Department of agriculture, land reform and rural development | The Micro Agricultural Financial Institutions of South Africa (MAFISA) | To facilitate the provision of equitable and large-scale access to financial services by economically active rural poor communities on an affordable, diversified, and sustainable basis |
| Department: Rural Development and Land Reform | National Rural Youth Service Corps (NARYSEC) | Is a youth flagship programme of the Department of Rural Development and Land Reform. It also provides character building programmes, soft and hard skills training and dispatch the youth to rural areas to undertake various rural infrastructure and other development projects. |
| FAO, 2020 | Digital agriculture profile; South Africa | To incorporate digital solutions into the agriculture industry of South Africa as it has displayed potential of addressing issues faced by this industry in South Africa. Digital solutions have the potential to create more efficient agricultural value chains by decreasing costs, increasing decision support, reducing loss, and improving sustainable resource use efficiency. |
| Food, Agriculture and Natural Resources Policy Analysis Network (FANRPAN), 2012 | Current and Emerging Youth Policies and Initiatives with a Special Focus on Links to Agriculture South African Case Study Draft Report | The aim of the policy is to conduct a Country Case Study (Literature Review) and present the findings of the current and emerging policies as well as related initiatives regarding the mainstreaming of youth development in the agricultural value chain in South Africa. Moreover, it is to identify space and opportunities for developing national ‘youth and agriculture policies’ within agricultural policies and make appropriate policy recommendations. |
| Mohokare local municipality | Draft youth development plan, Mohokare local municipality | To empower the youth of Mohokarane Local Municipality by constructing required and necessary interventions reduce the challenges that young people face such as unemployment. |
| National Youth Development Agency (NYDA) | Integrated youth development strategy (IYDS) (2021 – 2023) | An integrated, holistic, and sustainable approach towards youth development that contributes to the growth and development of South Africa’s youth. |
| National Youth Development Agency (NYDA), 2011 | The Integrated Youth Development Strategy (IYDS) of South Africa  2012- 2016 | The Aim of the IYDS is to accelerate the attainment of sustainable livelihoods and global competitiveness through mainstreamed and integrated youth development in all spheres of government, the private sector and civil society. |
| NEPAD, 2003 | Comprehensive Africa Agriculture Development Programme (CAADP) | To promote interventions that best respond to the widely recognised crisis of African agriculture. It aims to encourage African countries to pursue economic growth through agriculture-led development to reduce poverty and hunger on the continent. |
| Republic of South Africa Presidency, 2015 | The National Youth Policy (NYP) for 2015–2020 | The policy aims to create integrated, holistic, and sustainable youth development, conscious of the historical imbalances and current imbalances and current realities, to build a non-sexist, non-racist, democratic South Africa in which young people and their organizations not only enjoy and contribute to their full potential in the social, economic and political spheres of life but also recognize and develop their responsibilities to build a better life for all. |
| The Department of trade and industry | The National Youth Economic Empowerment Strategy and Implementation Framework Mainstreaming Youth in the South African Economy 2009 – 2019 | The strategy aims to promote and accelerate youth economic empowerment in the economy by prioritising an approach that encourages mainstreaming of the youth in the already existing core programmes designed for industrial and enterprise development. It also encourages and allow the formulation of new economic development programmes uniquely designed to target the youth only in circumstances where such an approach will either effectively address the existing deficiency or may have a much higher impact in empowering young people. |
| The Department of trade and industry | Youth Enterprise Development Strategy 2013-2023 | The vision of the YEDS is to create South Africa that has a dynamic youth entrepreneurship culture and developed youth-owned enterprises, operating in all sectors of the economy, which contribute to economic growth, poverty reduction and employment creation as well as assist in bringing about economic transformation and an equitable society. |
| The South African National Department of Agriculture in consultation with the  The FAO, 2003 | Agricultural Education and Training (AET) strategy for agriculture in rural development in South Africa | The aim of the AET strategy for Agriculture in Rural Development in South Africa is to address AET holistically in a manner that focuses all role players around three strategic goals. Moreover, it is to be accessible, responsive, quality Education and Training for Agriculture and Rural Development |

Table S3: Reasons for exclusion for articles removed at full screening stage

| **Author and year** | **Title** | **Reasons for exclusion** |
| --- | --- | --- |
| Mayer et.al (2011) | Towards a youth employment strategy for South Africa | Literature review, very limited primary data. |
| Mtembu & Govender (2015) | Perceptions of employers and unemployed youth on the proposed youth employment wage subsidy incentive in South Africa: A KwaZulu-Natal study | The main aim of the study was to determine the perceptions of unemployed South African youth and employers on the proposed youth wage subsidy incentive scheme. This is not related to the scope of the study. |
| Ngubeni (2015) | Youth development performance management in municipalities: A Nkangala district municipality case study | Literature review, no primary data |
| Silliman et.al, (2020) | Long-term effects of youth work internship: The Project Youth Extension Service approach | This study examines long-term effects of a youth work internship on participants’ current work performance. This is not related to the scope of the study. |
| Swarts & Aliber (2013) | The ‘youth and agriculture’ problem: implications for rangeland development | Literature review, no primary data |
| Trivelli & Jorge (2021) | Rural Youth Inclusion, Empowerment, and Participation | Literature review, no primary data |
| Wynveen &  McMahan (2019) | Youth Engagement in Forest Planning: Fulfilling the 2012 Planning Rule | The study was conducted in the United States and is not relevant to the South Africa. |
| Wilkinson et.al, (2017) | The employment environment for youth in rural South Africa: A mixed-methods study | The aim of this study was to identify factors associated with employment for out-of-  school youth in rural Mpumalanga, South Africa, and to help identify promising strategies to increase employment and reduce HIV vulnerability. This is not directly related to the scope of the study. |
| **Strategies, reports, and policy documents** | | |
| Department of Public Works, 2017 | Guidelines for the Implementation of the National Youth Service Programme under the Expanded Public Works Programme (EPWP) | The programme objectives are not directly related to the objectives of the study. Also, the document only includes the guidelines for implementation. |
| Department of Rural Development and Land Reform, n.d | National Rural Youth Service Corps programme | The full document of this policy is unavailable on the SA government website. A summary of the program is available at [www.gov.za](http://www.gov.za), [www.publicsectorleaders.co.za](http://www.publicsectorleaders.co.za). Authors made numerous unsuccessful attempts to contact the department to obtain the document with full details of the policy. |
| Department of Rural Development and Land Reform, n.d | Recapitalisation and Development Programme (RADP) | The full document of this policy is unavailable on the SA government website. Only the implementation evaluation report is available at <https://evaluations.dpme.gov.za/evaluations/407>. Authors made numerous unsuccessful attempts to contact the department to obtain the document with full details of the policy. |
